# Informal Caregiving Between Residents in LTC Facilities in India: Demanding or Rewarding Role

**DOI:** 10.1093/geroni/igab046.939

**Published:** 2021-12-17

**Authors:** Shantha Balaswamy

**Affiliations:** The Ohio State University, Columbus, Ohio, United States

## Abstract

Research on caring for older adult with health problems by Informal caregivers (IC) in the community in developing countries like India is increasing. However, IC in institutions is largely unacknowledged. This exploratory study examines the perceived role, demands, and rewards of informal caring for residents in independent LTC facilities in South India. A total of 187 residents were interviewed in Tamil and Kannada using structured and open-ended questions on demographics, health, mental health, residents’ interactions, tasks performed and personal experiences. About 50% reported assisting other residents with ADLs, 30% helped with IADLs, and 75% provided emotional support. The caregivers’ appraisals as residents and their relationship with care-recipient was both positive and negative. In addition to socialization, personal accomplishment, caregivers reported emotional exhaustion, stress, and burnout. Implications related to paid and unpaid labor policy in LTC and reducing IC stress are discussed.

